# Inhibitory Effect of *Nelumbo nucifera* (Gaertn.) on the Development of Atopic Dermatitis-Like Skin Lesions in NC/Nga Mice

**DOI:** 10.1155/2012/153568

**Published:** 2012-02-09

**Authors:** Rajendra Karki, Myung-A Jung, Keuk-Jun Kim, Dong-Wook Kim

**Affiliations:** ^1^Department of Oriental Medicine Resources, College of Natural Science, Mokpo National University, 61 Muan-gun, Jeollanam-do 534-729, Republic of Korea; ^2^Jeollanamdo Institute of Natural Resources Research, Jangheung-gun, Jeollanam-do 529-851, Republic of Korea; ^3^Department of Clinical Pathology, Taekyeung College, Gyeungsan, Gyeongsangbuk-do 712-719, Republic of Korea

## Abstract

Atopic dermatitis (AD) is a chronic inflammatory skin disease which has a complex etiology that encompasses immunologic responses. The study was carried out to examine the effect of *Nelumbo nucifera* (Gaertn.) leaf (NL) on the AD-like skin lesion induced by repeated epicutaneous application of 2,4-dinitrochlorobenzene (DNCB) on the dorsal skin of NC/Nga mice. Three different doses of NL (5, 25, and 50 mg/mice/day) were administered orally from the day of sensitization with DNCB for 4 weeks. The efficacy of NL was judged by histopathological examination, blood IgE level, measurement of transepidermal water loss (TEWL), scratching behavior, and skin severity score. NL resulted in the suppression of clinical severity score, TEWL, scratching behavior, and blood IgE level. Histopathologic analyses revealed that thickening of the epidermis and mast cell degranulation was significantly reduced in NL group. These results suggest that NL may be a useful natural resource for the management of AD.

## 1. Introduction

Atopic dermatitis (AD) is a chronic and relapsing inflammatory skin disease relying on the interplay of environmental, immunological, and genetic factors [1]. The prevalence of AD has increased dramatically in the past 3 decades and the increase in recent years can be attributed to environmental influences including industrialized and urban settings [2]. Epidemiological reports suggest that AD affects up to 10–20% of children worldwide and can persist into adulthood. It has been estimated that AD symptoms are developed among 65% of patients in the first year of life and among 90% of the patients before the age of five [3]. The lesional skin in AD is characterized by edema, hemorrhage, erosion, dryness, and alopecia typically localized on the ears, back, and neck and in the facial region [4]. Pathological examination of the lesional skin in AD reveals spongiosis, hyperkeratosis, and parakeratosis in acute lesions and marked epidermal hyperplasia, acanthosis, and perivascular accumulation of lymphocytes and mast cells in chronic lesions [5]. Although the complex interrelationships between genetic, environmental, skin barrier, pharmacological, psychological, and immunological factors contribute to the pathogenesis of AD, the immunological basis of the disease is of considerable importance and has been extensively studied [6]. The allergic diseases like AD, asthma, and rhinitis are characterized by Th2-dominated responses, which are mediated by IL4, IL-5, and IL-13 and induce B-cell class switching to IgE [7]. NC/Nga mice were established as an inbred strain from Japanese fancy mice in 1957 and have recently been shown to spontaneously develop AD-like dermatitis with IgE hyperproduction in air-uncontrolled, conventional circumstance [8, 9]. NC/Nga mice, however, show no skin lesion when raised in specific-pathogen-free (SPF) condition. An epicutaneous application with chemical antigens such as picryl chloride evokes contact hypersensitivity reaction in mice that had previously been sensitized with same agents. Interestingly, repetition of the topical application with such agents not only induces a shift in kinetics of the skin reaction from delayed-type to immediate-type response but also changes the cytokine milieu from Th1 to Th2 profile [10, 11].

*Nelumbo nucifera* Gaertn. (Nymphaeaceae), a large aquatic herb widely found in India, China, Japan, and Korea, not only possesses ornamental and dietary value but also has been using as a medicinal herb in eastern Asia. Almost every part of *N. nucifera* including leaves, flowers, seeds, and rhizomes has been reported to possess different therapeutic effects [12]. The leaves have been mentioned to show antiobesity activity by increasing lipolysis in the adipose tissue of mice [12, 13]. There are reports on *N. nucifera* rhizome possessing acetylcholinesterase activity and semen possessing antidiarrheal and tranquilizing activities [14]. *N. nucifera* is rich in alkaloids and flavonoids. Moreover, *N. nucifera* leaves contain abundant amount of flavonoids like luteolin, quercetrin, and isoquercitrin [15]. In this paper, the effects of water extract of *N. nucifera* leaves (NLs) on the slink skin symptoms of NC/Nga mice caused by the repeated topical application of DNCB (2, 4-dinitrochlorobenzene) were evaluated.

## 2. Materials and Methods

### 2.1. Animals

Male 5-week-old NC/Nga mice (20–23 grams body weight) were purchased from SLC Inc. (Tokyo, Japan). The mice were kept in standard plastic cages under controlled temperature (25 ± 5°C), humidity (50 ± 10%), 100% fresh hepa-filtered air, and 24 hours light-dark cycle (lights on from 06:00 to 18:00) in the Animal Research Center of Mokpo National University. The mice were supplied with basal diet and sterilized water without any restriction during the experiment. All mice were acclimated for a week prior to the initiation of experiments. All animal studies were approved by the Animal Care Committee of the Graduate School of Natural Sciences, Mokpo National University, and all husbandry practices and animal care were in accordance with the guidelines of Korean Council on Animal Care.

### 2.2. Reagents

DNCB was purchased from Sigma-Aldrich, Inc. (St. Louis, MO, USA). Olive oil and acetone were purchased from Kanto Chemical Co., Inc. (Tokyo, Japan) and Carlo erba Reagents SA. (France), respectively. DNCB was dissolved in olive oil/acetone (3 : 1) as 1% (w/v) and 0.4% (w/v) solution and used for the sensitization and elicitation.

### 2.3. Application of DNCB

Dorsal hair of NC/Nga mice was removed by using electric shaver and hair removing cream containing 80% of thioglycolate. On the next day, the dorsal skin was sensitized with 200 *μ*L of 1% DNCB (w/v) in olive oil/acetone (3 : 1). Four days later, the mice were challenged by applying 200 *μ*L of 0.4% DNCB (w/v) on the dorsal skin repeatedly 3 times per week (Monday, Thursday, and Saturday) for 4 weeks. Each group contained 7 mice.

### 2.4. Preparation of *Nelumbo nucifera* Leaf (NL) Extract

Leaves of *N. nucifera* were obtained from Muan, Korea. The leaves (1 Kg) were extracted with 3000 mL of distilled water by using soxhlet extractor for 3 hours, and the extraction was repeated for 3 times. The residue was removed by filtration and then the filtrate was evaporated and freeze-dried to give the powder of NL. The yield of the dried extract was about 12% of the starting material.

### 2.5. Administration of NL

NL was dissolved in distilled water and fed to the mice per orally using gastric sonde from the day of sensitization until 4 weeks. The control was fed with distilled water. NL was administered at 3 different doses (5, 25, and 50 mg/mice/day).

### 2.6. Transepidermal Water Loss (TEWL)

TEWL on the dorsal skin of mice was measured using a skin evaporative water recorder (Tewameter TM300; Courage and Khazawa, Cologne, Germany). The resulting data were analyzed by the microprocessor and were expressed in g/m^2^/h. TEWL was measured in each week for 4 weeks. Measurements were recorded when TEWL readings were stabilized at approximately 30 seconds after the probe was placed on the skin.

### 2.7. Evaluation of Scratching Behavior and Blood IgE Level

Mice were placed individually in a clear plastic cage and allowed to acclimatize for 15 minutes. Thereafter, behavior was videotaped for 10 minutes. Scratching of the nose, ears, and dorsal skin with the hind paws was observed during playback. Licking of the belly and dorsal skin during grooming was disregarded. Each occurrence of scratching of the head, neck, dorsal skin, ears, and nose was scored to obtain the maximum score. The IgE level in blood was measured using IgE kit (Shibayagi Co. Ltd., Gunma, Japan) according to the manufacturer's instructions.

### 2.8. Evaluation of Skin Severity

The severity of dermatitis on the face, ears, and dorsal part of the body was assessed blindly. The evaluated symptoms consisting of (i) erythema/hemorrhage, (ii) pruritus and dry skin, (iii) edema, (iv) excoriation/erosion, and (v) lichenification were scored as follows: none = 0; mild = 1; moderate = 2; severe = 3. The sum of the scores for each evaluated symptom (maximum score: 15) was considered as the skin severity score. The skin severity was evaluated every week for 4 weeks.

### 2.9. Histopathological Examination

After 4 weeks, all the mice were euthanatized under diethyl ether anesthesia and the dorsal skin was excised, fixed in 10% phosphate-buffered formalin (pH 7.2), and embedded in paraffin. Sections of 4 *μ*m were microtomed. The sections were deparaffinized in xylene, dehydrated in graded alcohol bath, and stained with hematoxylin and eosin or acidified toluidine blue. Finally, they were examined through optical microscope (Olympus, Tokyo, Japan), and thickness of epidermal layer was calculated using Aperio ImageScope image analysis. The number of mast cells per square millimeter of dermis in four sites chosen at random was counted from the toluidine stained sections.

### 2.10. Statistical Analysis

All the experimental data were expressed as mean ± standard deviation. The significance of variation among different groups was determined by one-way ANOVA analysis. *P* value ≤ 0.05 was considered to be significantly different.

## 3. Results

### 3.1. Effect of NL on Skin Severity

**Figure 1:**
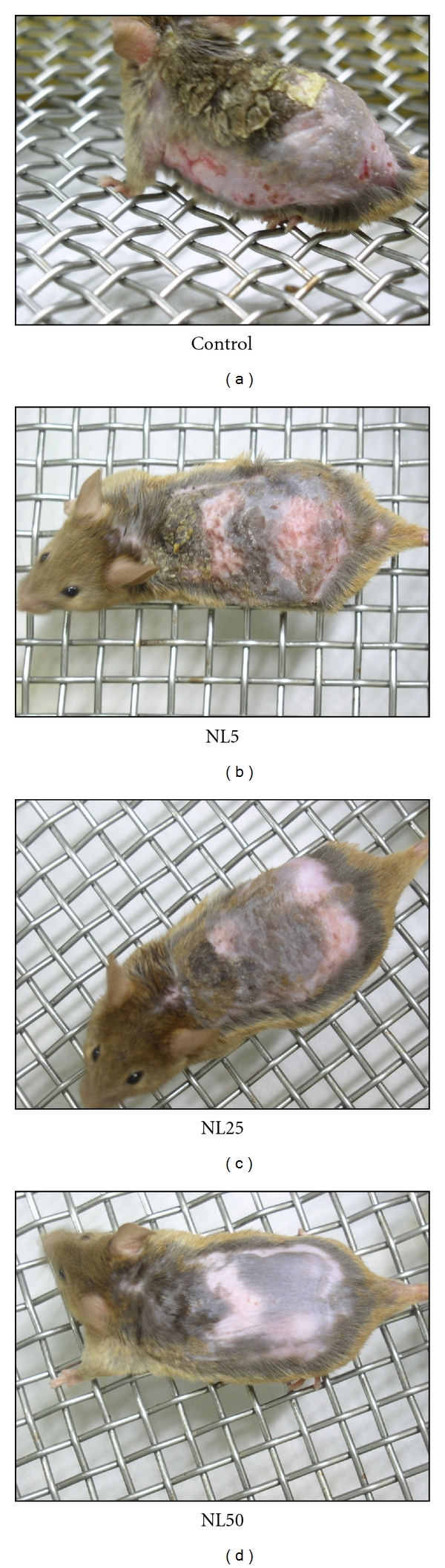
Clinical skin symptoms. Three different doses of NL (5, 25, and 50 mg/mice/day) were administered orally from the day of DNCB sensitization. The photographs were taken on day 28 before the sacrifice of the mice.

**Figure 2:**
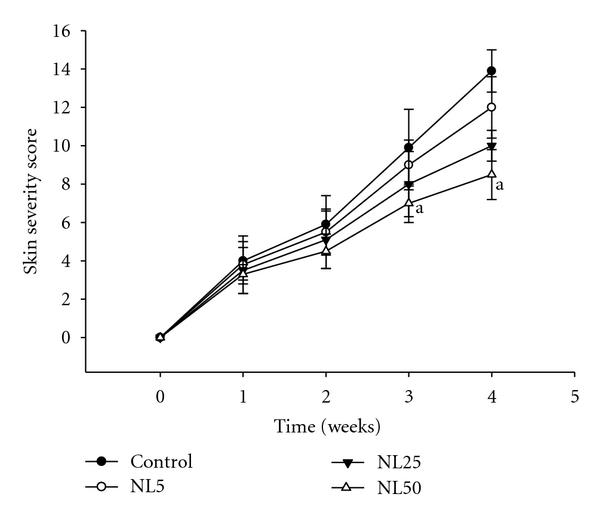
Skin severity score. The skin severity of mice was assessed macroscopically in a blinded fashion as mentioned in Section 2. The symptoms considered were (i) erythema/hemorrhage, (ii) pruritus and dry skin, (iii) edema, (iv) excoriation/erosion, and (v) lichenification. ^a^ *P*< 0.05 versus control.

NC/Nga mice have been shown to develop AD-like skin lesions by repeated application of picrylchloride. The skin severity in control was increased gradually with the number of DNCB challenges. Oral administration of NL for 4 weeks suppressed the development of AD-like skin lesions. The skin severity of each group on day 28 is as shown in Figure 1. Skin severity in each group was more or less similar up to 14 days from the day of sensitization. But after 14 days, there was drastic change in the skin symptoms of control than NL-administered groups. The skin severity score of each group is as shown in Figure 2.

### 3.2. Histopathological Analyses

**Figure 3:**
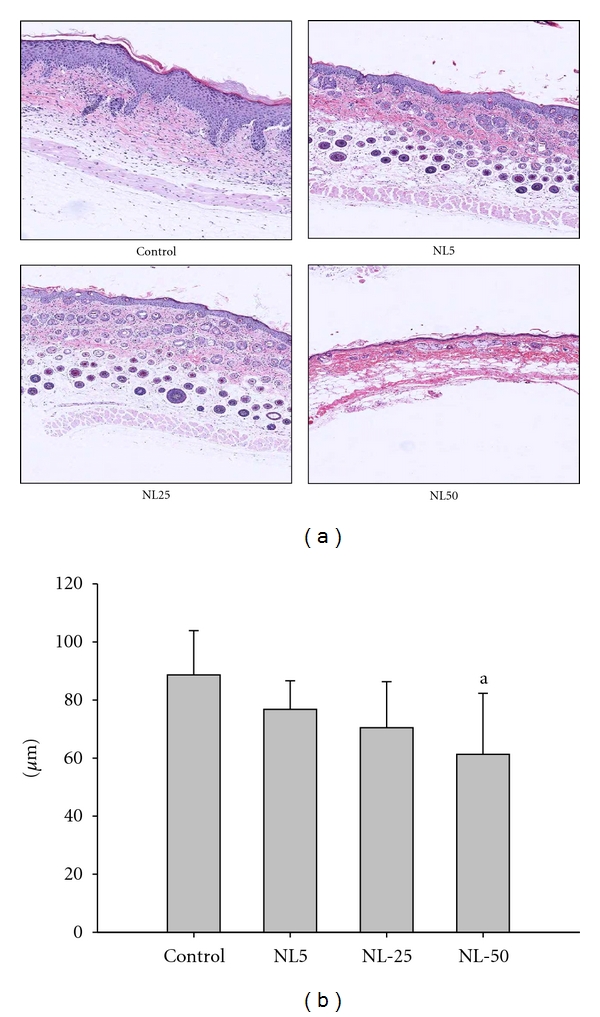
Histopathological changes. The dorsal skin excised at the end of the experiment was fixed in formalin, microtomed in 4 *μ*m sections, and stained with hematoxylin and eosin (magnification 400x). The thickness of epidermal layer was calculated using Aperio ImageScope V9.1.19.1571 image analysis. ^a^ *P* < 0.05 versus control.

**Figure 4:**
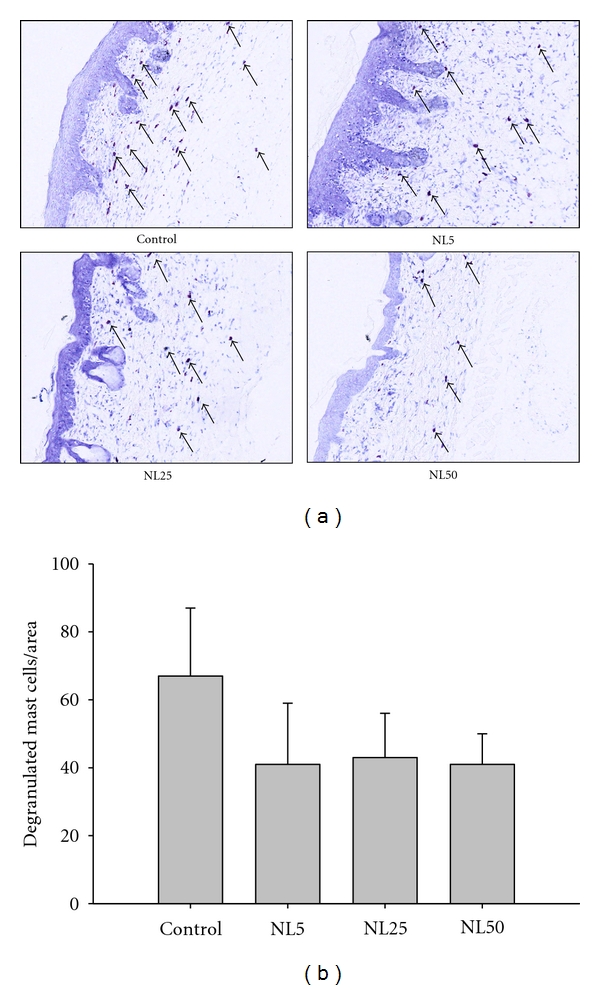
Mast cell degranulation. The dorsal skin excised at the end of the experiment was fixed in formalin, microtomed in 4 *μ*m sections, and stained with acidified toluidine blue. The number of mast cells/unit area of dermis in 4 sites chosen at random was counted. (magnification, 400x), arrow heads showing degranulated mast cells.

Hematoxylin and eosin staining of the dorsal skin sections revealed hyperkeratosis, parakeratosis, acanthosis with varying degrees of spongiosis, exocytosis of mononuclear cells in the epidermis, and infiltration of inflammatory cells into the upper dermis of control group while all those parameters were suppressed in NL-administered groups as shown in Figure 3(a). The quantitative data of epidermal thickness are as shown in Figure 3(b). The epidermal thickness of control was 88.668 ± 15.2 *μ*m while that of NL50 was 61.288 ± 21 *μ*m (*P* < 0.05). Toluidine blue staining of the dorsal skin sections revealed of more infiltration and degranulation of mast cells in the upper dermis of control than NL-administered groups as shown in Figure 4(a). The quantitative data on mast cell infiltration and degranulation are as shown in Figure 4(b). The number of mast cells per millimeter square in control, NL5, NL25, and NL50 was found to be 67 ± 20, 41 ± 18,  44 ± 13, and  41 ± 9, respectively.

### 3.3. Effect of NL on Blood IgE Level

**Figure 5:**
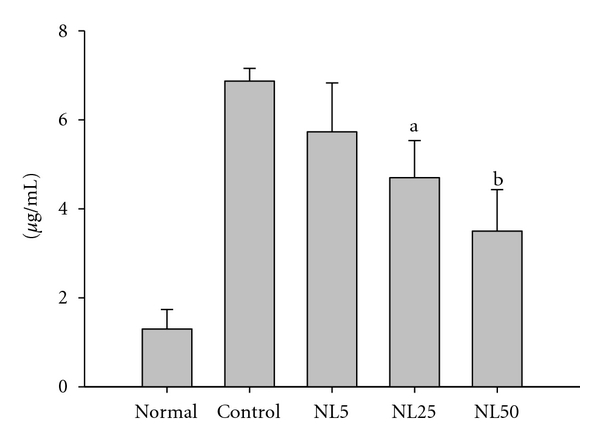
Blood IgE level. Blood was taken from each mouse at the end of the experiment, and the blood IgE level was measured using mouse IgE-specific ELISA kit. ^a^ *P* < 0.05 and ^b^ *P* < 0.01 versus control.

The epicutaneous sensitization and challenge of dorsal skin of NC/Nga mice with DNCB elevated the Ig E level from 1.36 ± 0.435 *μ*g/mL (normal, nontreated group) to 6.87 ± 0.286 *μ*g/mL. The administration of NL50 suppressed the IgE level to 3.5 ± 0.93 *μ*g/mL, which was the suppression approximately by 2 folds against the control. Similarly, NL25 suppressed the elevation of IgE to 4.7 ± 0.8 *μ*g/mL which was statistically significant (*P* < 0.05).  Figure 5 shows the effect of NL on the blood IgE level on day 28.

### 3.4. Effect of NL on Scratching Behavior and TEWL

**Figure 6:**
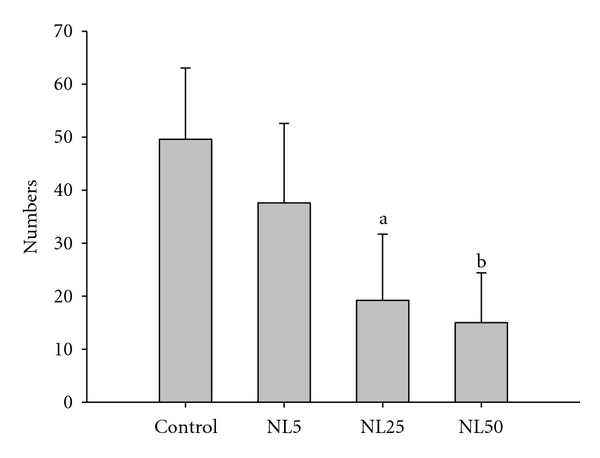
Effect of NL on scratching behavior. The scratching behavior of each mouse was videotaped for 10 minutes on day 25 after sensitization. Scratching of the nose, ears, and dorsal skin with the hind paws was observed during play back. ^a^ *P* < 0.05 and ^b^ *P* < 0.01 versus control.

**Figure 7:**
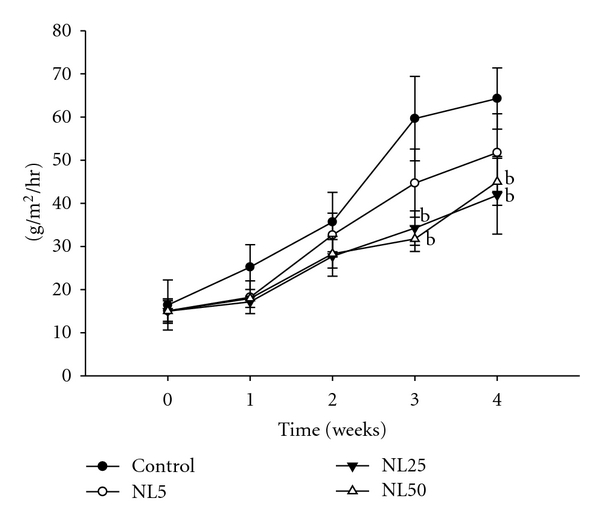
Effect of NL on TEWL. TEWL was measured every week for 4 weeks after sensitization using Tewameter (TM300). Measurements were recorded when TEWL readings were stabilized at approximately 30 seconds after the probe was placed on the skin. ^b^ *P* < 0.01 versus control.

There was no difference in the average number of scratching behavior among the groups up to 14 days. However, there were changes in the scratching behavior among the groups from the 3rd week. The scratching behavior evaluated on day 25 is as shown in Figure 6. The number of scratching was decreased by more than threefold in NL50. Consistent with effect of NL on mast cell degranulation, NL decreased the scratching behavior. TEWL of NL-administered groups was not different from control up to 14 days. However, administration of NL25 and NL50 significantly inhibited TEWL on days 21 and 28 (Figure 7).

## 4. Discussion

AD is a chronic inflammatory skin disease which has a complex etiology, and it encompasses immunologic responses, susceptibility genes, environmental triggers, and compromised skin-barrier function [1]. The skin lesions of AD patients are characterized by the presence of inflammatory infiltrates consisting of T lymphocytes, monocytes/macrophages, eosinophils, and mast cells [5]. Steroidal drugs like corticosteroids are commonly prescribed for the alleviation of the symptoms of AD. However, the repeated use of corticosteroids can cause severe skin atrophy, susceptibility to infection, and adrenal suppression [16]. NC/Nga mice, originated from Japanese fancy mice as an inbred line, whenever raised in conventional circumstances spontaneously develop AD-like skin lesions characterized by increased number of eosinophils, mast cells, lymphocytes, and macrophages [4]. Although SPF NC/Nga mice showed neither clinical signs nor IgE hyperproduction, application of haptens or mite antigens is necessary to evoke the eczema stably [17]. Consistent with these reports, epicutaneous sensitization and challenge of dorsal skin of NC/Nga mice with DNCB evoked the incidence of AD like skin lesions. Histological examination of skin lesion stained by hematoxylin and eosin and toluidine blue revealed the thickening of epidermis and dermis because of leukocyte infiltration, hyperplasia, dermis ulcer, and infiltration of immunocytes on the skin such as T cell, mast cell, and eosinophils. In NL-administered groups, the thickness of epidermis and dermis was reduced and the infiltration of immunocytes was significantly decreased as compared to control. Excessive production of chemokine such as thymus and activated-regulation chemokine (TARC) by activated keratinocytes attracts the immunocyte into the epidermis resulting in acanthosis [4]. In our *in vitro* study, treatment of NL (250 and 500 *μ*g/mL) for 24 hours significantly inhibited the production of TARC by activated keratinocyte (data not shown) suggesting that inhibition of TARC inhibited the infiltration of immunocytes thereby inhibiting epidermal thickening. Moreover, Th2-specific chemokines, TARC, and monocyte-derived chemotactic cytokine, and their receptor, CCR4, have been reported to be highly expressed in the lesions of the NC/Nga mice [4]. Haptens such as picryl chloride are commonly used to induce allergic dermatitis and have been thought to evoke primarily a Th1-dominated response. However, it has been recently reported that multiple challenges with picryl chloride to the skin of mice over an extended period cause the skin inflammation to shift to a chronic Th2-dominated inflammatory response that is similar to human AD [18]. It is well established that the elevation of serum IgE in AD may be due to the Th1/Th2 imbalance skewed to Th2, which plays important roles in the pathology of AD [19]. Mast cells, a critical participant in the various biological processes including allergic diseases and inflammatory reaction, store biologically potent inflammatory mediator such as histamine and express on their surface membrane receptors with high affinity and specificity for IgE [20]. The interaction of antigen with surface-bound IgE initiates a series of biochemical events that culminates in the release of histamine and the production of cytokines [21]. Histological examinations revealed that NL possessed the significant inhibitory effect on mast cell degranulation possibly due to inhibition of IgE hyperproduction. Pruritus, an unpleasant sensation provoking the desire to scratch in AD, might be due to histamine, proteases, and cytokines from various cells including degranulated mast cells [22]. In our study, NL inhibited the episode of scratching which might be due to inhibition of degranulation of mast cells. Xeroderma and skin barrier dysfunction, which are associated with AD, are due to lower level of ceramides that accelerate TEWL and decrease water capacitance resulting in atopic dry skin [23]. In our study, administration of NL inhibited TEWL thereby improving the skin condition. Although the hapten-repeated sensitization model is not a genetically driven model, many of its aspects may be applicable to extrinsic allergen-driven AD. In conclusion, we demonstrated that the oral administration of NL inhibited the development of AD like skin lesions in NC/Nga mice induced by repeated epicutaneous sensitization of the dorsal skin by DNCB. NL contains abundant amount of flavonoids like luteolin, quercetrin, and isoquercitrin [15] as pharmacologically active components. The anti-inflammatory effect of those flavonoids suggests the possibility of their therapeutic efficacy in various inflammatory diseases [24]. Furthermore, oral administration of NL for 4 weeks had no remarkable toxic effects such as reduction of food intake and body weight. Thus, the results from our experiment suggest that NL can be a potential natural resource for the management of AD although the mechanism of action involved in the treatment remains to be explored.

## 5. Conclusions

The present study provides evidence that oral administration of *N. nucifera* leaf extract attenuated the DNCB-induced atopic dermatitis like skin lesions in NC/Nga mice. Although we studied the effect of NL in chemical antigen-induced model, many of its aspects may be applicable to extrinsic allergen-driven AD.
